# On the nature of eye-hand coordination in natural steering behavior

**DOI:** 10.1371/journal.pone.0242818

**Published:** 2020-11-23

**Authors:** Jordan Navarro, Emma Hernout, François Osiurak, Emanuelle Reynaud

**Affiliations:** 1 Laboratoire d’Etude des Mécanismes Cognitifs (EA 3082), University Lyon 2, Lyon, France; 2 Institut Universitaire de France, Paris, France; Tongii University, CHINA

## Abstract

Eyes and hand movements are known to be coordinated during a variety of tasks. While steering a vehicle, gaze was observed to be tightly linked with steering wheel angle changes over time, with the eyes leading the hands. In this experiment, participants were asked to drive a winding road composed of bends with systematically manipulated radii of curvature, under regular and automatic steering conditions. With automatic steering, the vehicle followed the road, but the steering wheel and participants hands did not move. Despite the absence of physical eye-hand coordination in that condition, the eye and [what the hands should have done] to produce the action on the steering wheel were found to be coordinated, as under regular steering. This result brings a convincing piece of evidence that eye movements do more than just guiding the hands. In addition, eye-hand coordination was also found to be intermittent, context and person-dependant.

## 1. Introduction

*“Man owes a good part of his intelligence to his hands*” Jean Piaget. It is a truism to say that a large number of our daily activities are mediated by our hands, from switching the light on, making coffee, getting dressed, writing, to steering a car, catching a ball, etc. If not the only source of motor control, vision is largely involved in hand movements control (e.g. [1]). As a consequence, it comes with little surprise that the relationships between hand and eyes movements have been widely investigated for a variety of visuomotor tasks. For a long time, the focus of interest has been set on the quite complex control of simple reaching and grasping actions (e.g. [2–4]). A third type of eye-hand coordination has also been under the scope of investigations: manipulation.

Contrary to reaching and grasping, manipulation implies not only an action toward an object but also an interaction with that object or device that lasts for a period of time ranging from a few seconds to a few hours. The temporal coupling of eye-hand coordination during manipulation is dependant of the nature of the task (e.g. [5–10]). Despite this task dependency, common observations have been made across experiments: eyes fixations are (a) stereotyped, (b) most of the time directly relevant to the task, and (c) tightly linked in time to manual actions, more specifically preceding actions by up to a second [11]. It has been argued that eyes fixations precede hand movements in order to gather visual information “*just in time*” for guiding hand movements [12]. Such proactive eye fixations have been recorded in a variety of natural behaviours including reading music [6,13], typing [14], throwing basketball [15], putting in golf [10] or driving [16].

Eye-hand coordination is flexible so as to be adjusted to different tasks, at least when it comes to manipulation. Learning a quite complex eye-hand coordination (i.e. novel bi-manual mouse-like tool to move a cursor in order to hit successfully targets displayed on a screen) can be achieved in no more than 20 minutes. Such learning proceed in three consecutive stages, characterized by (stage 1) poor performances and the gaze following the cursor with occasional glances to the targets, (stage 2) performances increase and eyes continue to track the cursor but progressively tend to lead the cursor, and (stage 3) performances continue to improve gradually with the gaze directed toward the next target and no more to the cursor [17]. At stage 3, one can consider that an effective new eye-hand coordination, comparable with those observed in a variety of natural behaviours (e.g. reading music, typing, etc.), in the sense that eyes precede motor actions, has been learned. Once learned, proactive eye fixations in eye-hand coordination is robust and remains almost unchanged, even with a more complex task associated with performances impairment [18].

In their seminal field experiment [16] not only gathered information about where drivers are looking at while steering along a winding road, they also shed light on the eye-hand coordination engaged in such a driving situation (see also [19]). When driving on a single lane, drivers were reported to look at the right road edge on right-hand bends and at the left road edge on left-hand bends. Interestingly, horizontal gaze angle and steering wheel angle changes over time appeared to be very similar. Indeed, the shape of those two angles (eye and hands mediated by a steering wheel) was remarkably similar although with different scales and eyes were leading the steering wheel by about .8s. This relation has been interpreted as the steering wheel being turned at the angle corresponding to the eyes angle with a brief delay. This delay was understood as a safety margin, an anticipation useful in case a glance at a location irrelevant for steering (e.g. pedestrian, road sign, other vehicles, etc.) was made. Similar observations were made under another natural driving experiment ([20]; with a delay between eyes and steering wheel of about 1s) and a simulated driving experiment with bends radii of 200 or 300 meters, where the eyes and steering wheel angles were found to be highly correlated (*r* = .86) with the horizontal gaze angle leading the steering wheel angle by about 1.23s [21].

In sum, there is no doubt that in trained eye-hand coordination, the eyes are leading the hands in time. When it comes to car driving, such eye-hand coordination could be considered as part of the driving skills that are learned in order to steer the car efficiently (operationnal control: [22,23]). The eyes are leading the hands not only in time but also by providing critical information directly useful for the motor control of hands movements. This interpretation has been favoured for long as drivers are looking at a specific dynamic location of the road (i.e. the tangent point) that is geometrically linked to the radius of curvature of the bend [16,11]. Thus, looking at that particular location of the road would allow drivers to appreciate the curvature of the road and to use directly eyes angle to adjust steering angle. Moreover, when drivers were asked to fixate a spot at the centre of the screen, preventing the natural eye-hand coordination to appear, driving performances were dramatically impaired. This result was interpreted as an evidence of eyes guiding hands: “*it is the appropriateness of what the eyes are doing that determines performance*” ([24], p. 418). If driving performances are impaired when the eyes cannot move freely, what would happen when the eyes can move freely, the vehicle steered efficiently, but with the hands remaining unmoving?.

The objective of the current work was to investigate the nature of eye-hand coordination under regular steering circumstances (i.e. the participant is in charge of steering with the visual driving scene available) as opposed to an automatic steering of the vehicle (i.e. the participant is not in charge of steering with the visual driving scene available). In the regular steering condition both eyes and hands can move freely, thus participants could implement the well-known eye-hand coordination specific to car driving. In the automated steering condition, the vehicle followed the path along the road, but the steering wheel and participants hands remained still. If participants were free to look at the dynamic driving scene associated to the vehicle, no eye-hand coordination was possible since the hands remained on an unmoving steering wheel. By breaking the relationship between the eyes and the hands, we hypothesize that eye movements under the automatic condition would no longer be devoted to eye-hand coordination but rather to visual exploration of the driving scene.

Another objective was to test the eye-hand coordination flexibility through a systematic manipulation of the road curvature and its analysis depending on bends sections.

## 2. Method

### 2.1. Participants

Eighteen participants (8 females and 10 males), aged from 18 to 29 years old (mean = 24 ± 2.9) took part in the experiment. They had 2.8 to 11.9 years of driving experience (mean = 5.4 ± 2.6) and declared an average of 12 833 kilometres (± 23365) driven in the last 12 months. The study was approved by the Ethics Committee of the Department of Psychology of Lyon, and informed consent was obtained from all participants. All participants had normal or corrected vision and none experienced motion sickness.

### 2.2. Materials

#### 2.2.1. Driving simulation

**Fig 1:**
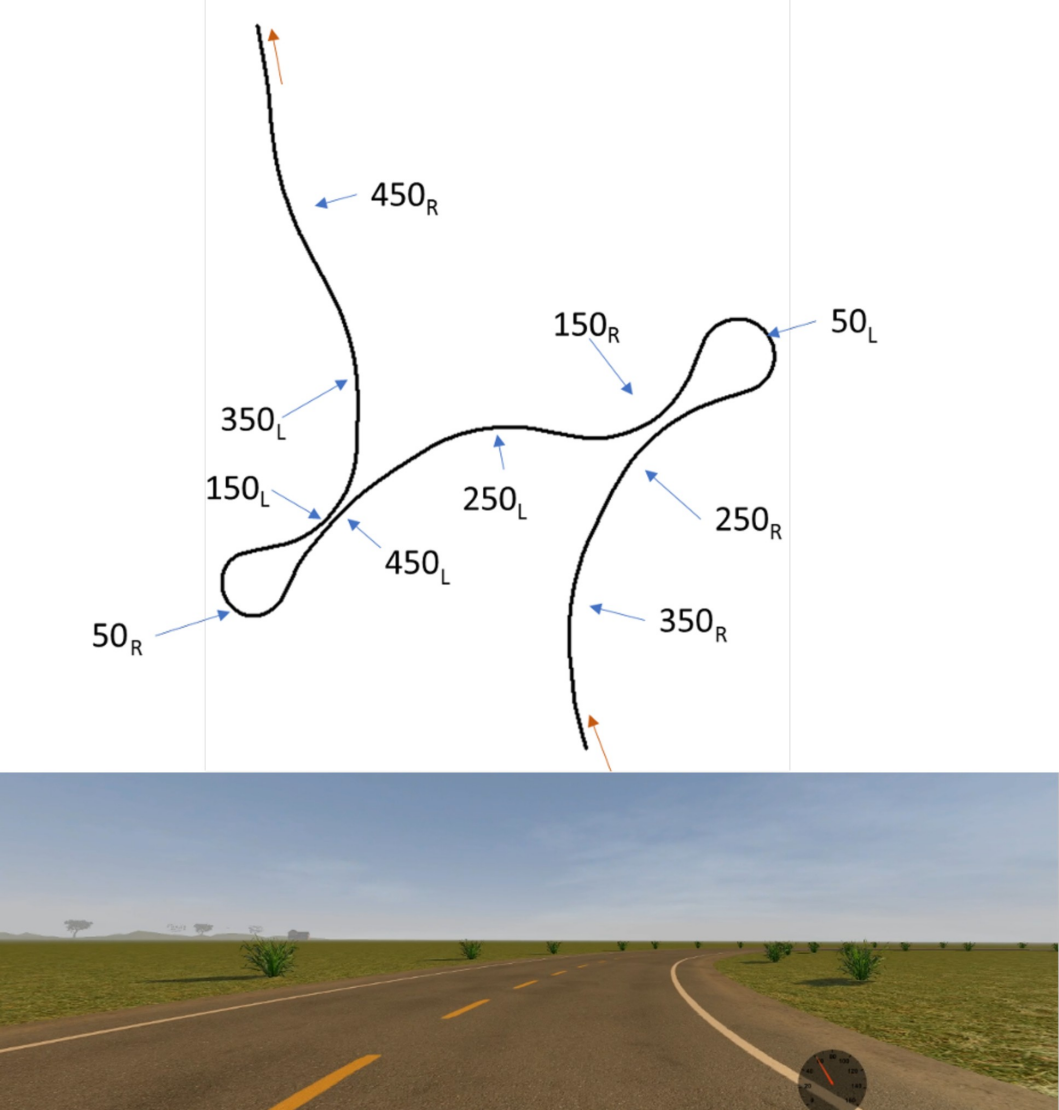
Top: Layout of the track. The red arrows indicate the driving direction, the blue arrows point to the different bends and associated direction (L: Left and R: Right) and radii of curvature in meters. Bottom: Screenshot of the simulated road used for the experimental drives, note that the width of the image was cropped (Photograph JN).

The experiment was carried out on a fixed-based driving simulator composed of an adjustable seat (JCL®), three 22-inch screens offering a visual angle of about 138° in the horizontal and 29° in the vertical axis and steering wheel with force feedback, accelerator and brake pedals Logitech G27®. An automatic gearbox was used, and a speedometer was displayed at the bottom of the visual scene. The driving simulation was developed at the University of Sherbrooke; see [25] for more details about the driving simulation. A visual driving environment was developed for the purpose of the experiment and consisted in a series of bends on a two-lane main road with bends (Fig 1). The track was composed of 10 bends of 200 meters of length, each bend being separated from the other by 50-meters straight lanes. Out of the 10 bends 5 were on the right and 5 on the left. Each bend had a specific constant radius of curvature (50, 150, 250, 350 or 450 meters). The track started with a 100-meters straight section before the 10 bends of 200 meters each, separated by 9 straight lanes of 50 meters, and finally 100 meters to stop after the winding bend, for a total of 2650 meters.

#### 2.2.2. Eye movement tracker

Drivers’ gaze behaviors were recorded by means of a gaze tracker (Iview X head-mounted, Sensomotoric Instruments®) at a sample rate of 50 Hz and using a most accurate 9-points calibration procedure.

### 2.3. Procedure

First, participants signed the informed consent, then they were equipped with the eye-tracker. Next, participants drove a familiarization drive, steering along a road with a variety of left and right bends, until they declared to be at ease with driving on the simulator. The familiarisation drive usually lasted about 10 minutes. The road used for the familiarization drive was different from the road used for the experimental drives. After familiarization, participants completed two identical drives, one under Regular Steering (RS) condition and the other under Automated Steering (AS) condition. The calibration of the eye-tracker was checked and adjusted if required before each drive. The order of presentation of the two drives was counterbalanced between participants. Under AS, participants were told that they were seated in an autonomous vehicle perfectly reliable, as such they had no action to perform on the vehicle controls (i.e. steering wheel or pedals). No further information was given to participants but to keep monitoring the driving environment. Under RS participants were asked to drive as they would do if driving in real life.

### 2.4. Data analysis

**Fig 2:**
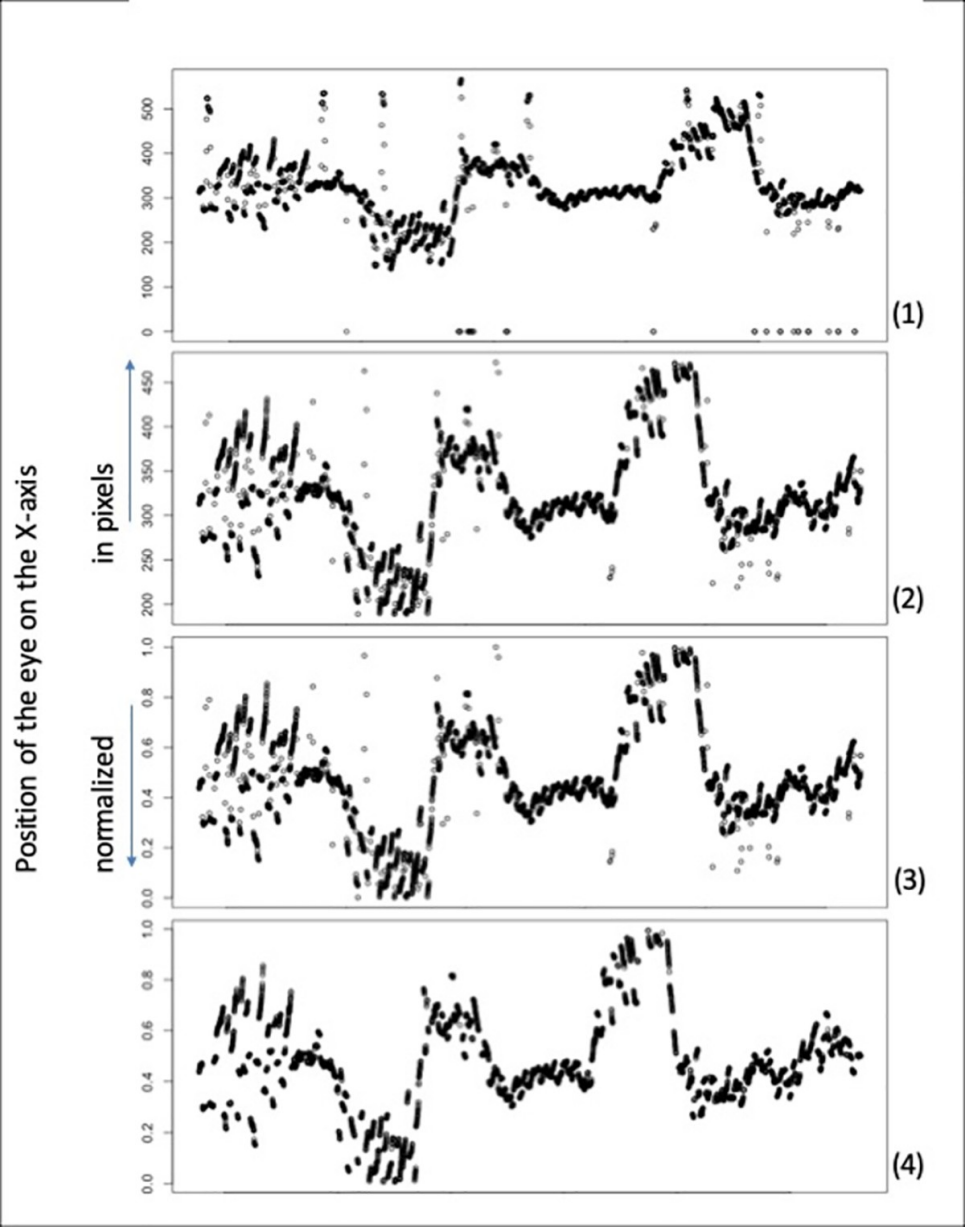
Example of the preprocessing used on the eye data, for a single participant under Regular Steering (RS). (1) raw data of the eye position on the X-axis, (2) eye position after preprocessing step a: removal of blinks and outliers (mean ± 2 SD filter), (3) eye position after preprocessing step b: normalization, (4) eye position after preprocessing step c: saccades removal.

Raw eye movement data were pre-processed in three steps: (a) outliers, including blinks, were removed by eliminating any points located over two standard deviations from the mean, (b) the data were normalized between 0 and 1 for each drive of each participant and (c) saccades were removed (eye movements faster than 1.3 cm or 20 pixels per second^-2^ were considered as saccades). About 7% of the data (including blinks) were removed at pre-processing step a, and about 15% at pre-processing step c. An example of a sample of raw and pre-processed data is presented on Fig 2. Raw steering wheel data were normalized and downgraded to a sample rate of 50Hz (original sample rate of 60Hz) to be synced with eye data. Under AS, if the steering wheel remained unmoved for the complete duration of the drive, steering wheel angles corresponding to the changes of vehicle heading were still provided by the driving simulator.

**Fig 3:**
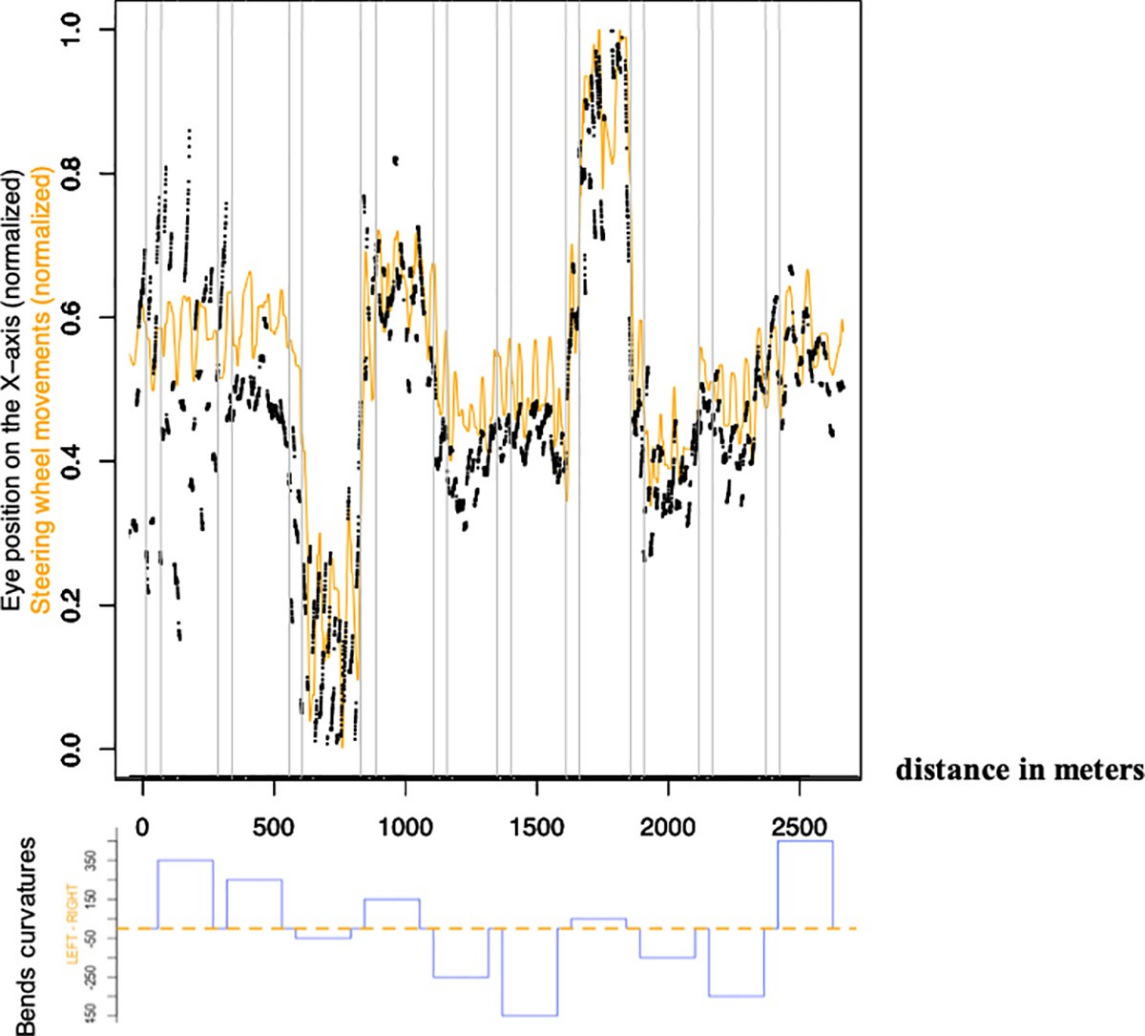
Top: Example of the eye position on the X-axis (in black) and steering wheel movements (in orange), both normalized, along the 10 bends and 9 straights lines for a single participant under Regular Steering (RS). Bottom: bends curvatures along the road for the complete drive (Up = Right bend, Down = Left bend).

Several analyses were then undertaken using pre-processed horizontal eye movements and steering wheel data (see Fig 3 for an example). First, eye and steering wheel data were correlated from the beginning of the first bend to the end of the last bend (Pearson’s correlation). A pairwise *t*-test was used to compare mean eye-steering wheel correlations under RS and AS. RS and AS conditions mean eye-steering wheel correlations were also plotted for the different participants.

Second, the time series from eye and steering wheel data were cross-correlated over a period of time corresponding to 100-time steps (equivalent to ± 2 seconds) to estimate the time lag between the eyes and the steering wheel angle. A pairwise *t*-test was used to compare RS to AS time lags between eyes and steering wheel. The time lags between RS and AS conditions were also correlated for the different participants.

Third, running correlations, operating on a sliding window of 4 seconds, were computed between the eyes and the steering wheel signals. These running correlation allows to explore the variation of the correlation between eyes and hands over time. It was computed for (1) each complete bend, (2) for the first half of each bend -i.e. the first 100 m- and (3) for the second half of each bend -i.e. the last 100 m-.

Repeated measures ANOVAs with three factors (steering condition [Regular Steering, Automatic Steering], the radius of curvature [50, 150, 250, 350, 450 meters] and bend direction [Left, Right]) were performed for all three running correlation measures between the eyes and the steering wheel (complete bends, first half of the bends and second half of the bends). Finally, a pairwise *t*-test was used to compare the mean running correlation in the first and second half of the bends.

Tukey HSD tests were used for post-hoc comparisons and a level of significance of *p* < .05 was used in all tests.

## 3. Results

### 3.1. Eye-steering wheel coordination

#### 3.1.1. Eye-steering wheel correlation

**Fig 4:**
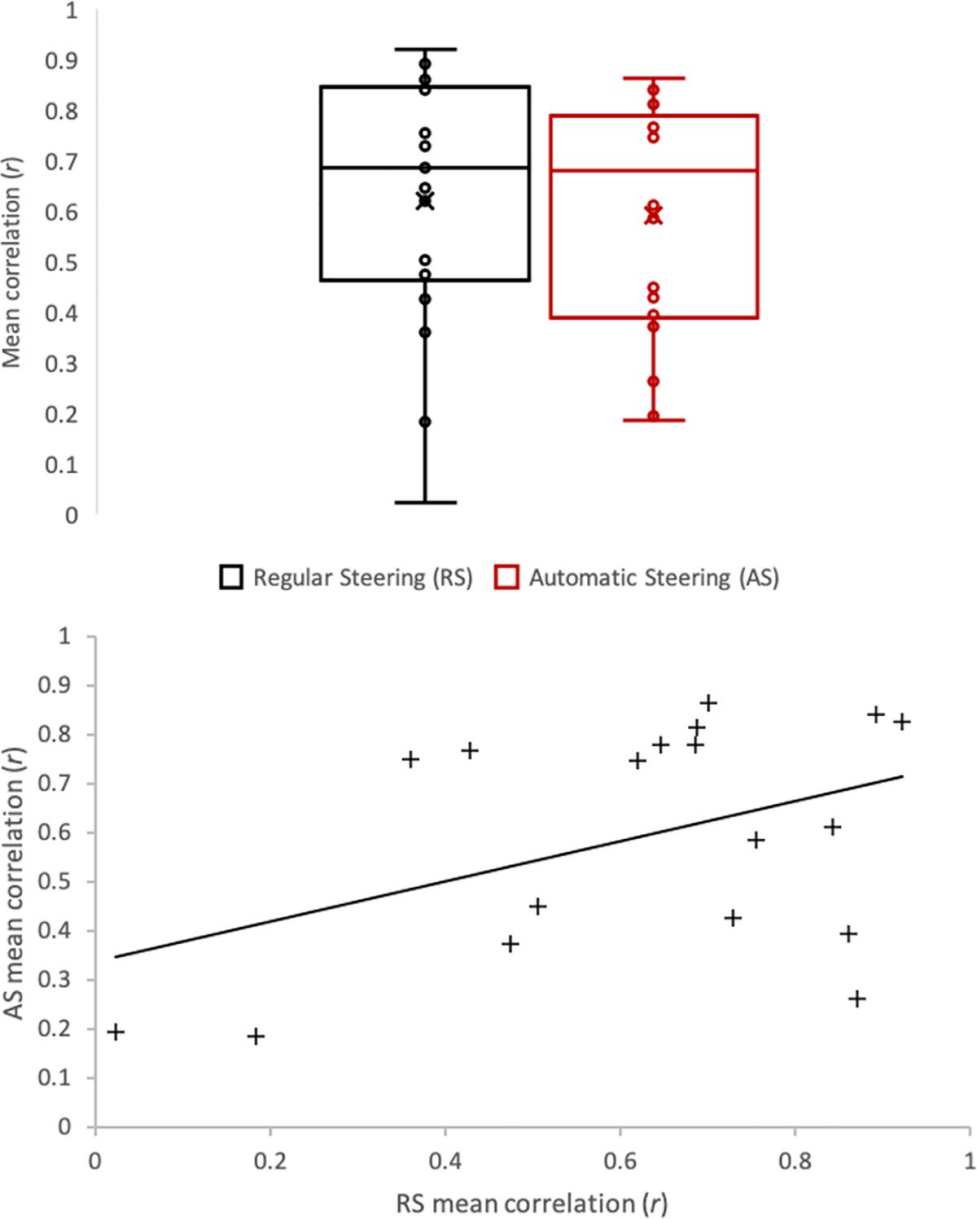
Top: Box plots representing mean eye-steering wheel correlations distributions, from the beginning of the first bend to the end of the last bend, in RS and AS conditions. Circles represent participants mean eye-steering wheel and the cross the average. Each box is composed of three horizontal lines (the middle one represents the median and the two others represent the first and the third quartile) and minimums and maximums outside the first and third quartiles are depicted with whiskers. Bottom: Linear correlation between RS and AS mean eye-steering wheel correlation. Each cross represents a participant.

Fig 4 (top) represents mean eye-steering wheel correlations distributions over the complete succession of the 10 bends and 9 straight lines for RS and AS. Mean eye-steering wheel correlations collected under RS were not significantly different from those collected under AS (*t*(17) = .47; *p* = .68). As depicted on Fig 4 (top) by the circles representing the different participants, inter-individual differences in mean eye-steering wheel correlations have been recorded.

Mean individual eye-steering wheel correlations have been found to be correlated in RS and AS conditions (*r* = .43; *p* < .04; Fig 4, bottom), revealing a consistent individual pattern of eye-steering wheel correlation between the two steering conditions.

#### 3.1.2. Time lag between eyes and steering wheel

**Fig 5:**
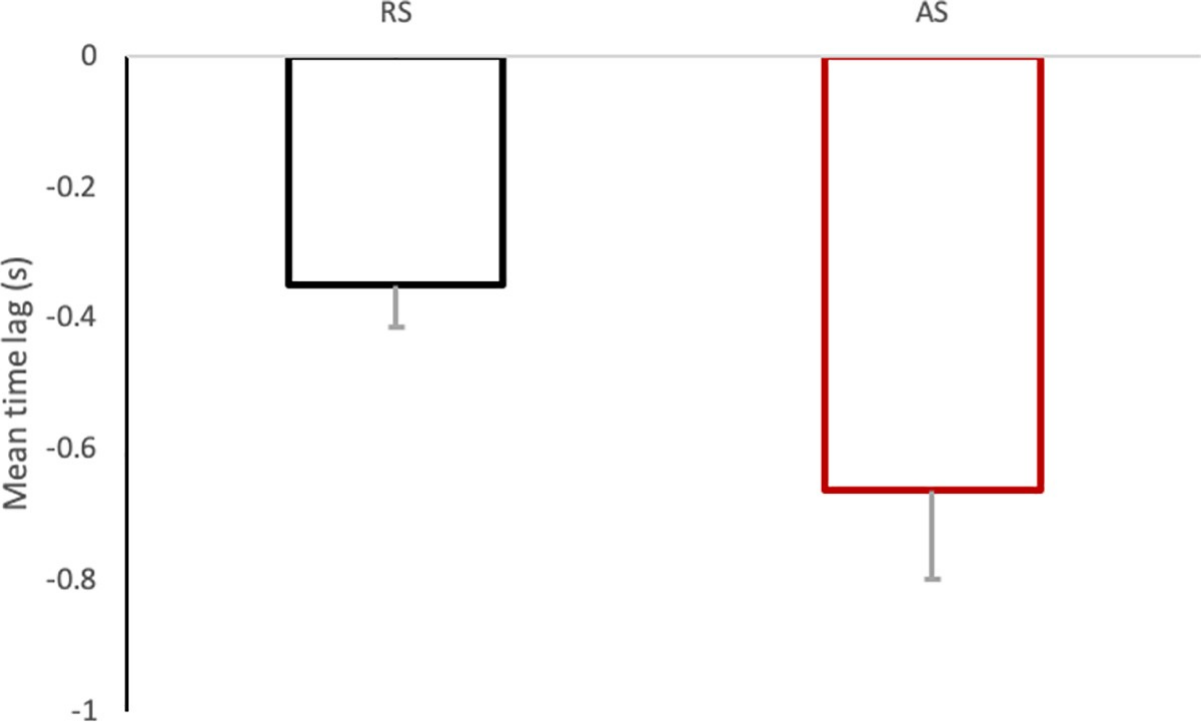
Mean eye-steering wheel time lag for RS and AS conditions. A negative value indicate that eyes lead steering wheel. Error bars represent one standard error.

The mean time lag between the eyes and the steering wheel was found to be significantly different between RS and AS (mean time lag RS: -.25s and AS: -.66s; *t*(17) = 2.33; *p* < .02; Fig 5). The individual correlation between RS and AS did not reveal an individual significant pattern of time lag between eyes and steering wheel (*r* = .31; *p* = .11).

### 3.2. Running correlation between eyes and steering wheel

#### 3.2.1. Complete bends

**Fig 6:**
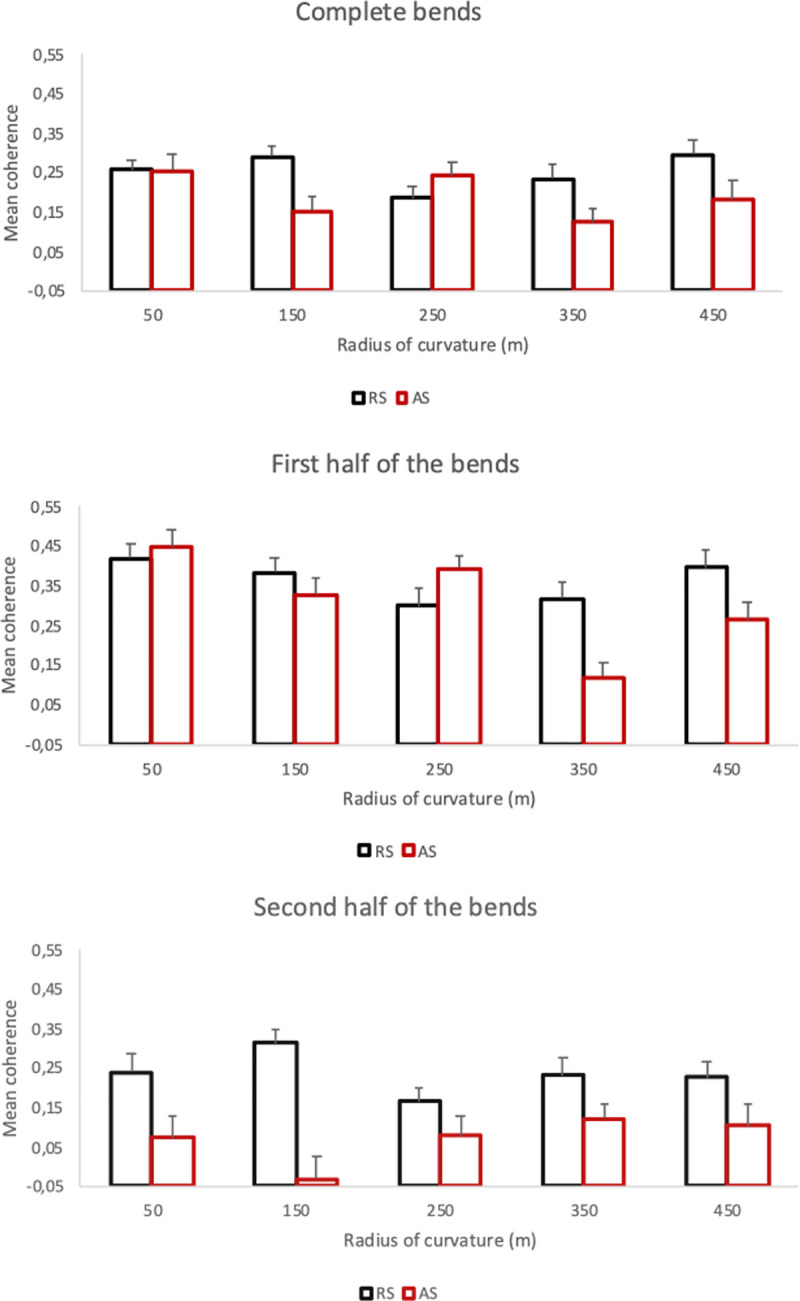
Mean running correlation depending on the steering condition (RS: Regular Steering; AS: Automatic Steering) and the radius of curvature of the different bends (50, 150, 250, 350 or 450 meters); for complete bends (left), first half of the bends (middle) and second half of the bends (right). Error bars represent one standard error.

The ANOVA showed a main effect of both the steering condition (*F*(1,17) = 22.59, *p* < .001, *η*^2^ = .053) and the radius of curvature (*F*(4,68) = 3.44, *p* < .02, *η*^2^ = .33) but no main effect of the bend direction (*F*(1,17) = .12, *p* = .73) nor interaction between steering condition and radius of curvature (*F*(4,68) = .70, *p* = .59; see Fig 6 left).

Regarding the main effect of steering condition, a mean running correlation of .253 was recorder under RS as compared to a mean of .145 under AS. Regarding the main effect of the radius of curvature, post-hoc revealed a significant difference between the bends with a radius of curvature 50, 150 and 450 meters (mean running correlation: .205, .245 and .244 respectively) and those with a radius of curvature of 250 and 350 meters (mean running correlation: .151 and .150 respectively).

#### 3.2.2. First half of the bends

The ANOVA showed a main effect of the radius of curvature (*F*(4,68) = 22.56, *p* < .02, *η*^2^ = .03) but no main effect of the steering condition (*F*(1,17) = .01, *p* = .91), the bend direction (*F*(1,17) = .75, *p* = .40) nor interaction between steering condition and radius of curvature (*F*(4,68) = .91, *p* = .46; see Fig 6 middle).

Regarding the main effect of the radius of curvature, post-hoc revealed a significant difference between the bends with a radius of curvature of 50 meters (mean running correlation: .451) and those with a radius of curvature of 250 (mean running correlation: .326) and 350 (mean running correlation: .294).

#### 3.2.3. Second half of the bends

The ANOVA showed a significant main effect for the steering condition (*F*(1,17) = 7.20, *p* < .02, *η*^2^ = .04), radius of curvature (*F*(4,68) = 3.73, *p* < .01, *η*^2^ = .03), but no significant effect of the bend direction (*F*(1,17) = .04, *p* = .84), nor interaction between steering condition and radius of curvature (*F*(4,68) = .75, *p* = .56; see Fig 6 right).

Regarding the main effect of the steering condition, a mean running correlation of .253 was recorded under RS as compared to a mean of .125 under AS. Regarding the main effect of the radius of curvature, post-hoc revealed a significant difference between the bends with a radius of curvature of 150 meters (mean running correlation: .246) and those with a radius of curvature of 250 (mean running correlation: .104).

#### 3.2.4. First versus second half of the bends

The mean running correlation was higher in the first half (.36±.17) compared to the second half (.18±.11) of the bends (*t*(17) = 7.66; *p* < .001).

## 4. Discussion

In the reported experiment, the focus was set on the previously documented eye-hand coordination engaged while steering a vehicle along a winding road [19,16,21,24,26,27]. Here, the coordination between eyes and hands was manipulated through the use of an Automatic Steering condition (AS) where the vehicle automatically moved along the road without any movement on the steering wheel. In that condition, participants were free to explore a driving scene, equivalent to the one available under Regular Steering (RS), but with the steering wheel and the hands remaining motionless. The mean correlation between eyes and steering wheel angles was found equivalent under RS and AS conditions, even though steering wheel angle was not physically available to participants in the AS condition.

Such a result is counterintuitive as eyes movements have repeatedly been described as guiding the hand in a variety of visuo-motor tasks including steering a vehicle (e.g. [19,28–30]). Moreover, with vehicle steering automation, further ahead visual scanning of the driving environment were repeatedly recorded, while driving on a rural road [31], avoiding obstacles [32] or following another vehicle [33]. However, it is reported here that the eye [what the hands should have done to produce the action on the steering wheel] are still coordinated. If this result is not ruling out the usefulness of eyes movements for manual action control, it brings a convincing piece of evidence that eye movements do more than just guiding the hands. This result is questioning the nature of eye-hand coordination.

The persistence of a physically impossible eye-hand coordination (in the AS condition) is in line with the idea that eye movements serve to supply a feedforward model of hands movement (associated to the steering wheel angle and ultimately vehicle direction) to come, as proposed by engineering models for long [34,35] and observed experimentally [36,37]. The reported results favour the idea that during steering, eye movements are anticipatory, feedforward and open loop [38,39]. Eye movements may feed an internal forward model of the steering control, found to be supported by the cerebellum [40], and consistent with a meta-analysis of steering control while driving [23].

However, the physically impossible eye-hand coordination observed did not rule out the important role of visual feedback in steering control [41–43]. Indeed, participants might have used visual information, not only to anticipate, but also to assess the actions of the automatic controller in the AS condition. This interpretation is supported by the important time lag increase in AS compared to RS (from -.349s in RS to -.662s in AS). The eye-steering coordination (even if physically impossible in AS condition) is much more anticipative under AS than under RS, as if participants did not change the eye-steering coordination but increased the eye movements anticipation when motor control of the steering wheel was not required. Such a strategy could be due to the disappearance of the time required to plan and execute motor actions and would allow participants to keep their eyes to the location required to adjust the steering wheel angle before the steering wheel angle change is required. As if participants tented to anticipate the automatic controller actions a little more than their own actions. This way participants could both anticipate and monitor actions of the automatic steering wheel angle changes under AS. In addition, participants could have used peripheral vision to ensure that the automatic controller was adjusting the lateral position of the vehicle efficiently, a task drivers are known to perform using peripheral vision cues [44]. A poor level of trust in automation, a key concept when considering human-machine interactions [45–47], including those related to highly automated driving (see [48] for a review) might explain the preservation of eye-hand coordination under AS. Indeed, it could be hypothesized that participants keep on scanning the visual environment under AS in a similar way to what they do under MD, because they did not completely trust automatic steering. Experiments lasting over an extended period of time, including measures of trust in automatic steering, would be required to investigate the relationships between trust in automatic steering and eye-hand coordination.

The reported data also favour the idea that eye-hand coordination is not an automated visuo-motor control loop but is rather flexible, not only between different tasks as already observed but also inside a given task. Indeed, eye-hand coordination has not only been observed when no motor actions were required, but eye-hand coordination was also found to be intermittent, context and person-dependant.

Steering a vehicle is often described as learning how to handle the car position in lateral and longitudinal dimensions (at least at an operational control level; [22]), thus a learner needs to build a robust specific coordination between physical actions on the vehicle control (i.e. steering wheel and pedals) and vehicle movements in the world assessed visually. In the dual processing theory, such eye-hand coordination is falling into the automatic processing category [49–51]. But, again eye-steering coordination was observed in AS even if physically impossible. This result indicates that the motor dimension of the coordination is not required to observe a tight link between eye movements and vehicle movements.

In addition, the eye-steering coordination was found to be intermittent. The running correlation between eye and steering wheel angle is strong during the first half of bends (i.e. first 100 m) but weakened during the second half of those same bends (i.e. last 100 m). This suggests an intermittent eye-hand coordination depending on the need to adjust the vehicle direction. The estimation of, and adjustment to, bend curvature during the first half of bends requires such coordination but to a lesser extent in the second half of bends. This observation is consistent with previous data revealing that if gaze often leads steering, the reverse (i.e. participants steering first and then look back to the road ahead) is also possible when performing a visual secondary task with low-eccentricity targets [52].

Moreover, eye-steering coordination was found to be dependant of the driving context, with a running correlation between eyes and steering wheel angles being influenced by the radius of curvature of the bends. Again, this observation is indicating that eye-steering coordination is flexible, in line with degraded eye-steering coordination when drivers were distracted [53], anxious [30], under the influence of alcohol [54], or instructed to gaze at a fixed spot (Marple-Horvat et al., 2005). To keep on improving the understanding of eye-hand coordination, such coordination should be investigated when the eye-hand coordination is disturbed unexpectedly, for instance in case of strong crosswind [55]. Finally, eye-steering coordination was found to be person-dependant. If inter-individual differences were already observed in several experiments (e.g. [6,20,30,53]), the current study revealed that the strength of eye-steering coordination is consistent at an individual level between the RS and AS conditions, suggesting different individual preferences and/or strategies. Further studies are required to better investigate the potential relationship of the strength of the eye-steering coordination with driving performances and other individual characteristics such as expertise or personality for instances.

The reported experiment would also greatly benefit from further investigations to overcome some of its current limitations. Indeed, in the current experiment the focus was set on eye-hand (through steering wheel) coordination, but while driving, it is not only required to steer the vehicle. Driver also needs to control the vehicle speed, look for potential hazard (e.g. other vehicles, pedestrian) and navigate in the road network among other tasks [56]. Here, a very simple steering task without traffic or traffic signs and very little scenery was proposed. In that way, it was expected that participants would focus on the steering task. Still, eye movements would probably be different in a more realistic context. In addition, it would be valuable to focus not only on eye-movements direction but also on the locations participants gaze at in the driving environment, although these analyses are challenging in dynamic environments such as driving [57]. Experiments over an extended period of time, in particular for the AS condition, would also lead to a better understanding of the eye-hand coordination, allowing to investigate a potential unlearning process of eye-hand coordination as opposed to the known learning process [17].

## 5. Conclusion

Eye-hand coordination (through steering wheel) is believed to allow people to efficiently steer their vehicle along winding roads in their everyday lives. The current experiment was designed to refine the understanding of the nature of eye-hand coordination in natural steering behaviour. The main finding is that eye-hand coordination was observed even when removing the motor component of that visuo-motor coordination. The persistence of a physically impossible eye-hand coordination favours the idea that eye movements serve to supply a feedforward model of vehicle direction. Eye-hand coordination was also found to be intermittent, as well as context and person-dependant. In other words, eye-hand coordination is under the dynamic influence the environment (position on the bend and bend radius of curvature) but is also sensitive to individual strategies.
